# A New Taraxastane Triterpene from *Euphorbia Denticulata *with Cytotoxic Activity Against Prostate Cancer Cells

**Published:** 2018

**Authors:** Sara Shamsabadipour, Seyed Mohammad Zarei, Mustafa Ghanadian, Seyed Abdulmajid Ayatollahi, Mohammad Reza Rahimnejad, Hojjatollah Saeedi, Mahmoud Aghaei

**Affiliations:** a *Department of Biology, Faculty of Science, University of Isfahan, Isfahan, I.R. Iran. *; b *Phytochemistry Research Center, Shahid Beheshti University of Medical Sciences, Tehran, I.R. Iran. *; c *Isfahan Pharmaceutical Sciences Research Center, Isfahan University of Medical Sciences, I.R. Iran.*; d *Biochemistry Department, Isfahan Faculty of Pharmacy and Pharmaceutical Sciences, Isfahan University of Medical Sciences, Isfahan, I.R. Iran.*

**Keywords:** *Euphorbia denticulate*, Triterpenes, Cycloartane, Taraxastane, Prostate Cancer, Cytotoxicity

## Abstract

In this research, aerial parts of *Euphorbia denticulata* Lam were collected from Sanandaj city in Kurdistan province at the West of Iran. It was extracted by maceration using acetone as solvent. The isolation of compounds were carried out with repeated column chromatography using silica gel and normal preparative HPLC using YMC-Pack-Sil column and hexane: ethyl acetate as mobile phase. The structures of isolated compounds were elucidated by extensive 1 and 2D-NMR as well as HRESI-MS spectra and the cytotoxicity was done against DU-145 human prostate cancer cell line using standard MTT assay. It afforded a new 12-taraxastane derivative and two known cycloartane triterpenes including: taraxast-12-ene-3β,20,21(α)-triol (1), cycloartane-3β,25-diol (2), and cycloartane-3β,24,25-triol (3). They exhibited cytotoxic effects, with IC_50_ values of 12.2 ± 2.9, 27.5 ± 4.9, and 18.3 ± 1.4 µM, respectively.

## Introduction

*Euphorbia *is one of the largest groups in angiosperm genera with about 2000 species. According to Rechinger, there are ninety six species in Flora Iranica classified into five sections, from which *Tithymalus* is the largest one in Iran. *E. denticulata* is a perennial herb, belonging to section *Tithymalus*, with expanded leaves without stipules, cyathia arranged in cymose rays around a cyathium and with large and denticulata glands in outer margins (1, 2). 

To the best of our knowledge, there is no specific application in folk medicine for *E. denticulata*. But, in general *Euphorbia* is used in treatment of gout وback pain, as a laxative and locally for treatment of warts and sores (3). Phytochemical studies have shown that *Euphorbia* is a rich source for different types of macrocyclic diterpenes, triterpenoids, and glucoseinulates. Triterpenes found in this genus include cycloartane, euphane, tirucallane, ursane, lupane, oleanane, and taraxastane structures. Among them, taraxastane type triterpenes are basically enantiomer of ursanes; differ mainly in H-18, H-29, and H-30 stereochemistry. They were interestingly reported to have remarkable multidrug resistance modulation, apoptosis induction of cancer cells, cytotoxicity against different cancer cells, α-glucosidase inhibitory activity, and antimicrobial activity (4-7).

In the previous researches done by the same authors on this plant a number of triterpenes and steroids including: botulin, 24-methylene-cycloart3-oL,cycloart-23Z-ene-3β,25-diol, cycloart-23Z-ene-3β,25-diol,ergosta-8, 24-dien-3-o, and beta-sitosterol were reported (8). In this paper, we have described the isolation, structure elucidation, and cytotoxic activities of three triterpenes from *E. denticulata*. Compound **1** is a new taraxastane derivative, compounds **2** and **3** are cycloartane type triterpene which are reported in *Euphorbia denticulate* for the first time. 

## Experimental

*General*

Isolation of compounds was carried out with open column chromatography using silica gel (60-200 μm, Merck, Germany) and normal preparative HPLC using a YMC-Pack-Sil column (250 × 20 mm i.d., YMC, Japan). The structures of the compounds were elucidated by ^1^H-NMR, BB ^13^C-NMR, DEPT, COSY, HMBC, NOESY, FT-IR and HRESI-MS. The NMR spectra were acquired with Bruker AV 400 using CDCI_3_ as solvent. The Infrared spectra were gained by Rayleigh WQF-510 FTIR spectrophotometer. The HRESI-MS spectra were obtained with Waters Q-TOF Micro YA019 mass spectrometer in *m/z*. 

*Plant material*

*E. denticulata* was collected from Sanandaj city in Kurdistan province in the West of Iran. It was identified by Professor Hojjatollah Saeedi in the Department of Biology, Faculty of Science, University of Isfahan and a voucher specimen number 19001 was deposited there in the herbarium of the University of Isfahan (Iran).

**Table 1:** ^1^H and ^13^C NMR Data of Compound 1 at 400 and 100 MHz in CDCl_3_

**Atom**	***δ***_H_ ** (** ***J*** ** in Hz)**	***δ***_C_
1	1.02 (1H, bdd, *J*=5.4, 18.5 Hz); 1.58 (1H, m)	37.2 (t)
2	1.49 (1H, m); 1.54 (1H, m)	27.7 (t)
3	3.18 (1H, dd, *J*=4.1, 11.2 Hz)	79.3 (d)
4	-	39 (s)
5	1.22 (1H, dd, *J*=5.4, 11.1 Hz)	50.6 (d)
6	1.42 (1H, m); 1.40 (1H, m)	18.2 (t)
7	1.37 (1H, overlapped); 1.73 (1H, m)	33.9 (t)
8	-	43.6 (s)
9	-	48.9 (d)
10	-	34.9 (s)
11	1.86 (1H, m); 2.04 (1H, m)	23.9 (t)
12	5.19 (1H, dd, *J*=2.8, 7.0 Hz)	117.8 (d)
13	-	145.8 (s)
14	-	33.9 (s)
15	1.16 (1H, overlapped); 1.61 (1H, m)	31.8 (t)
16	1.21 (1H, m); 1.85 (1H, overlapped)	28.5 (t)
17	-	29.7 (s)
18	1.40 (1H, overlapped)	53.5 (d)
19	1.39 (1H, m)	35.6 (d)
20	-	73.2 (s)
21	3.29 (1H, dd, *J*=5.0, 8.4 Hz)	78.5 (d)
22	1.31 (1H, m); 1.29 (1H, m)	28.7 (t)
23	0.9 (3H, s)	27.6 (q)
24	0.79 (3H, s)	14.7 (q)
25	0.67 (3H, s)	13.1 (q)
26	0.76 (3H,s)	22.1 (q)
27	0.91 (3H, s)	27.3 (q)
28	1.10 (3H, s)	23.2 (q)
29	0.78 (3H, d, *J*=6.2 Hz)	18.5 (q)
30	1.15 (3H, s)	29.6 (q)

**Figure 1:**
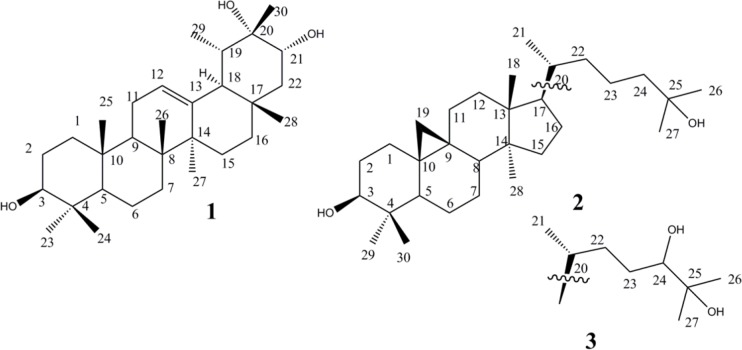
Chemical structures of compounds **1-3** isolated from* E. denticulata*

**Figure 2:**
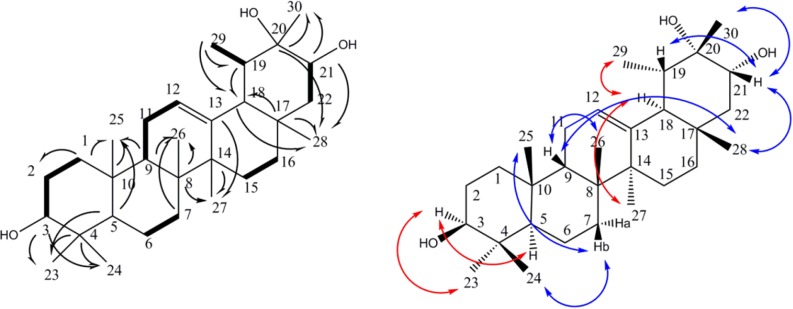
COSY (in bold), selected HMBC (H→C), and selected NOESY (H↔C) correlations detected for compound 1

**Figure 3:**
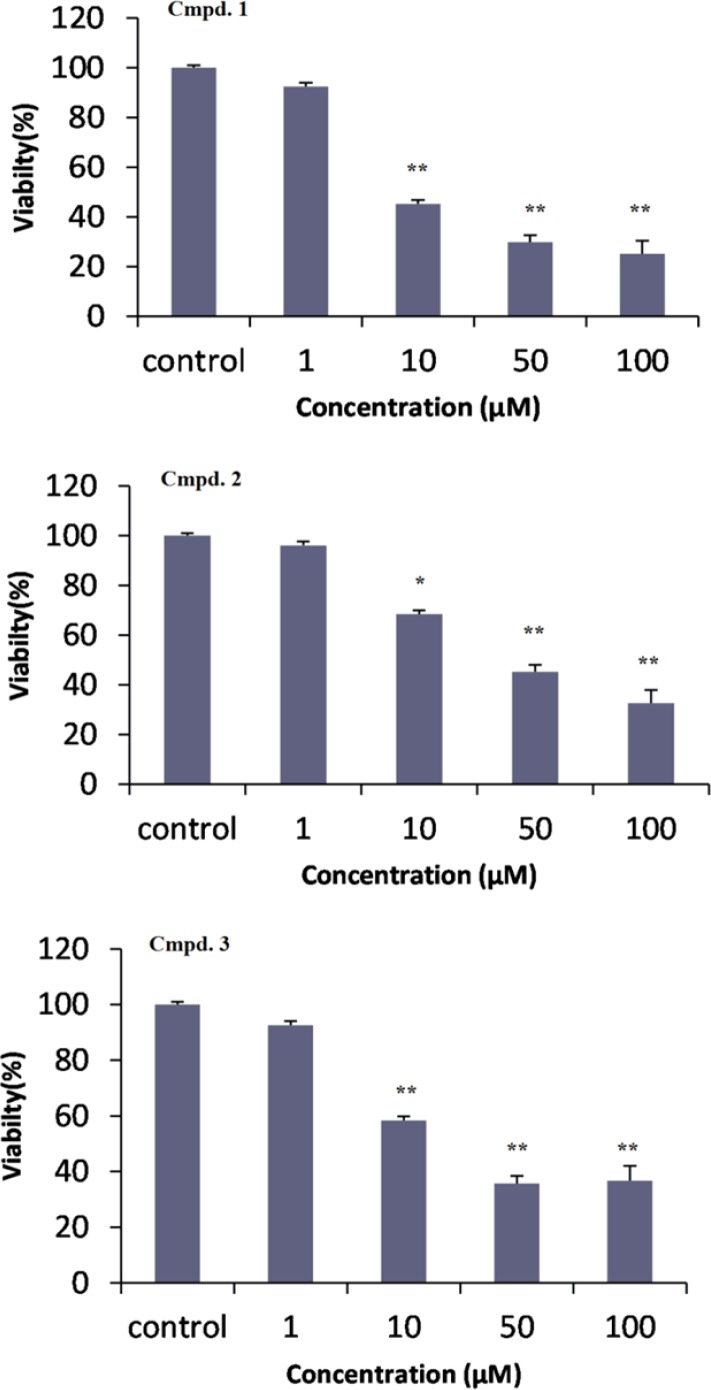
Cytotoxicity effects of compounds 1-3 against DU-145 human prostate cancer cells were treated with different concentrations (1, 10, 50, 100 µM) in three replicates. Results (mean ± SD) were calculated as percent of corresponding control values. (* *P* < 0.05; ** *P* < 0.001 versus control

*Extraction and isolation*

The air-dried plant material (1000 g) was macerated for three days with acetone (6 L×3). Filtration and in vacuum evaporation resulted in a green gum (85 g), which was applied on VLC over RP-18 (40-63 µm) using MeOH:H2O (7:3) as eluent. Then, defatted extract (12 g) was concentrated and column chromatographed on flash silica gel (40-63 µm, 200 g) using hexane/EtOAc, with increasing polarity (5→50 %) to afford eight fractions. Inspecting by ^1^H-NMR, Fr.2, Fr.3, and Fr.4 contained characteristic signals of steroids and triterpenoids which were previously reported from this plant by the same authors (8). None of the fractions showed characteristic resonances related to macrocyclic polyester diterpenes. Fraction Fr.5 eluted with hexane/EtOAc (75:25) was subjected to more purification on HPLC using YMC-Pak-Sil column (250 × 20 mm) and hexane/ EtOAc (80:20) as mobile phase to yield compound 1 (Fr.5a1, 7.6 mg), and compound 2 (Fr.5a2, 5.2 mg). Fraction Fr.8 eluted with hexane/EtOAc (60:40) was subjected to more purification on HPLC using hexane/ EtOAc (70:30) as mobile phase to yield compound 3 (Fr.8a1, 9.8 mg) (Figure. 1).

*Compound* ***1: ***Amorphous white powder, MW (g/mol): 458; yield: 0.001%; IR (KBr) υ_max_: 3430, 2945, 2870, 1640, 1465, 1145, 1106 cm^-1.^ ^1^H-NMR (CDCl_3_, 400 MHz): see Table 1; ^13^C-NMR data (CDCl3, 100 MHz): see Table 1. Positive HR-ESIMS *m/z* 459.3865 (calcd. for C_30_H_50_O_3_ + H^+^, 459.3833, Δ 7.0 ppm).

*Compound* ***2*** *: *Amorphous white powder, MW(g/mol): 444 yield: 0.0015%; ^1^H-NMR (CDCI_3_, 400 MHz): δ_H_ 3.32 (1H, dd, *J *= 4.2, 10.5 Hz, H-3), 1.25 (1H, s, H-27), 1.19 (1H,s, H-26), 1.00 (1H, s, H-18), 1.00 (1H, s, H-29), 0.92 (1H, s, H-28), 0.91 (1H, d, *J *= 6.2 Hz, H-21), 0.58 (1H, d, *J *= 4.2 Hz, H-19b), 0.36 (1H, d, *J *= 4.2 Hz, H-19a). ^13^C-NMR data (CDCl_3_, 100 MHz): 78.8 (C-3), 73.3 (C-25), 52.3 (C-17), 48.8 (C-14), 48.0 (C-8), 47.1 (C-5), 45.3 (C-13), 40.5 (C-4), 36.4 (C-24), 35.9 (C-20), 35.6 (C-12), 33.5 (C-22), 32.9 (C-15), 32.0 (C-1), 30.4 (C-2), 29.9 (C-19), 29.7 (C-7), 26.6 (C-27), 26.5 (C-16), 26.0 (C-10), 26.0 (C-11), 25.4 (C-30), 23.2 (C-26), 22.7 (C-23), 21.1 (C-6), 20.0 (C-9), 19.3 (C-28), 18.4 (C-21), 18.1 (C-18), 14.0 (C-29). Positive HR-ESIMS *m/z* 445.4054 (calcd. for C_30_H_52_O_2 _+ H^+^, 445.4040, Δ 3.1 ppm).

*Compound* ***3*** *: *Amorphous white powder. MW(g/mol): 460; yield: 0.0015%; ^1^H-NMR (CDCI_3_, 400 MHz): δ_H_ 3.37 (1H, dd, *J *= 5.3, 7.0 Hz, H-24), 3.31 (1H, dd, *J *= 4.4, 11.2 Hz, H-3), 1.24 (3H, s, H-27), 1.19 (3H,s, H-26), 0.99 (3H, s, H-18), 0.99 (3H, s, H-29), 0.92 (3H, s, H-28), 0.90 (3H, d, *J*=6.4Hz, H-21), 0.57 (1H, d, *J *= 4.1 Hz, H-19b), 0.36 (1H, d, *J *= 4.2 Hz, H-19a). ^13^C-NMR data (CDCl_3_, 100 MHz): 78.8 (C-24), 78.7 (C-3), 73.2 (C-25), 52.4 (C-17), 48.8 (C-14), 48.0 (C-8), 47.1 (C-5), 45.3 (C-13), 40.4 (C-4), 35.9 (C-20), 35.5 (C-12), 33.1 (C-22), 32.9 (C-15), 31.9 (C-1), 30.3 (C-2), 29.9 (C-19), 28.4 (C-23), 28.2 (C-7), 26.6 (C-27), 26.4 (C-16), 26.0 (C-10), 26.0 (C-11), 25.4 (C-30), 23.2 (C-26), 21.1 (C-6), 19.9 (C-9), 19.3 (C-28), 18.1 (C-21), 18.0 (C-18), 14.0 (C-29). Positive HR-ESIMS *m/z* 461.4012 (calcd. for C_30_H_52_O_3 _+ H^+^, 461.3989, Δ 4.9 ppm).

*Cytotoxicity MTT assay*

DU-145 human prostate cancer cell line from Pasteur Institute, Iran, was grown adherently in RPMI-1640 with 10 % fatal calf serum, 100 units⁄mL penicillin and 100 μg⁄mL streptomycin at 37 °C in 5% CO_2_. The cells were seeded at 5000 cells per well in 5% CO_2_ at 37 °C in RPMI with 10% FBS, 100 U/mL penicillin and 100 µg/mL streptomycin, in 96-well plates. After 24 h incubation, the cells had been treated with concentrations (1, 10, 50, and 100 µM) of compounds **1**-**3** for 2 days. MTT was added to the wells and incubated for another 4 hours. The experiment was done in triplicate and the absorbance was read by the microplate reader (Bio-Rad, Hercules, CA, USA) at 570 nm. Cell viability percentages calculated by the formula: (mean OD of treated cells /mean OD of control cells) ×100 and were expressed as percent of control cells which were not treated (9).

*Statistical analysis*

All data are reported as mean ± SD of the mean and the IC_50_ values were calculated using Excel based program. T-test with two sample assuming equal variances and Dunnet test one way ANOVA were used and *P*<0.05 was considered to indicate a statistically significant difference.

## Results and Discussion

Acetone extract is applied on VLC over RP-18 to remove nonpolar components and then column chromatographed on flash silica gel and purified on HPLC using normal Silica column to yield compound 1-3.

Compound **1** was obtained as a white powder. Its HR-ESIMS showed a positive pseudo molecular peak ion at *m/z* 459.3865 calculated for C_30_H_50_O_3 _+ H^+^ (459.3833, Δ 7.0 ppm). 1D and 2D-NMR spectra showed signals for eight methyls, eight methylenes, seven methines, and seven quaternary carbons, including two oxy-methine groups, one oxy-carbon, and an olefin bond. A total of 30 carbons were observed -characteristic of a triterpene skeleton. Application of ^1^H-^1^H correlation spectroscopy (COSY) allowed the multiplets of the triterpene structure to be sequenced into six spin systems: H-1 to H-3, H-5 to H-7, H-9 to H-12, H-15 to H-16, H-21 to H-22, and H-19 to Me-29 (Figure 2). Heteronuclear long range correlations (HMBCs) observed between Me-23/C-4, Me-24/C-4, Me-25/C-10, Me-26/C-8, Me-27/C-14, Me-28/C-17 allowed location of methyl groups C-23, 24, 25, 26, 27 and 28 (Figure 2). Key HMBC correlations exhibited by H-3/ C-23; H-5/ C-25, C-23; H-7/C-26; H-9/C-25, C-26; H-27/C-13, C-14; H-15/C-14; H-18/C-17; H-29/C-18; H-21/C-28, C-30 connected the spin systems and build up the whole structure as an taraxast-12-en triterpene skeleton. Finally, HMBC correlations of C-3/ Me-23, Me-24; C-21/Me-28, Me-30; C-20/ Me-30 as well as ^1^H–^1^H-COSY connectivity›s assigned two oxymethin resonated at *δ*_C_ 79.3 , and 78.5 ppm at C-3, and C-21, and quaternary oxygenated carbon (*δ*_C_ 73.2 ppm) at C-20 (Figure 2a). Stereochemistry of 1 was deduced from NOESY spectrum and literature data. Taking H-5 (α), NOESY correlation between H-3/ H-5, H-23; H-27/H-18; H-18/H-29 defined α orientation for these protons, while NOE cross peaks of Me-24/H-7b; H-7b/Me-25; H-9/Me-26; H-9/Me-28; Me-28/H-21; H-21/H-19; H-21/Me-30 supported *β* position for Me-24, Me-25, H-9, Me-26, Me-28, H-21, H-19, Me-30, respectively (Figure 2b). Regarding the above data, compound 1 was elucidated as 12-taraxast-3β, 20, 21 (α)-triol.

Comparison of the ^1^H- & ^13^C-NMR as well as mass spectral data with those published before allowed us to establish the structures of compound 2 and 3 as cycloartane-3β, 25-diol and cycloartane-3β, 24 (S),25-triol as known cycloartane type triterpenes. To the best of our knowledge, it is the first time that NMR data of cycloartane-3β, 25-diol are reported. It had been identified previously by HR-GCMS analysis alone from matured *Wheat straw* by Gaspar in 1992 (10). Compound **3**, cycloartane-3β, 24 (S) 25-triol, is isolated and reported for the first time from natural origin. It is semi-synthesized and reported before by Della Greca and coworkers after treating of 24, 25-epoxide-3 (β)-cycloartanol with methylmagnesium N-cyclohexylisopropylamide (11). Its isomer cycloartane-3β, 24 (R), 25-triol was also semi-synthesized and reported in the same manner by Anjaneyulu and the coworkers from alkaline hydrolyzing of 3β, 24 (R)-diacetoxy-cycloartane-25-ol (12).

To assess the effects of the isolated compounds on the viability of cancer cell lines, 12-taraxast-3β, 19, 21 (α)-triol (1), cycloartane-3, 25-diol (2) and cycloartane-3,24, 25-triol (3) were tested against DU-145 human prostate cancer cell line, *in-vitro* (9). Compounds 1 to 3 showed cytotoxic effects with IC_50_ values (the concentration that inhibited the cell growth by 50%) of (1):12.2 ± 2, (2): 27.5 ± 4.9, and (3): 18.3 ± 1.4 µM, respectively. All of three compounds (1-3) in the concentration of 10, 50, and 100 µM showed significant difference with control (*P* < 0.05). But, their cytotoxic effects were not significant in lower concentration of 1 µM (*P* > 0.05). In comparison between compounds 2 and 3 with similar cyloartane structure at concentration of 10 µM, compound 2 showed weaker cytotoxicity effect than compound **3** (*P* > 0.05).

## Conclusion

In this study a new 12-taraxastane derivative with three hydroxyl group located at C-3, C-19, and C-21 as well as two known cycloartane triterpenes were extracted from *E. denticulata*. Taraxastan triterpens are very rare in nature and it is the first report of their occurrence in endemic spurges of Iran. Compounds **1**, and **2** both showed potent cytotoxic activity against DU-145 human prostate cancer cell line, *in-vitro*. However, MTT assay could be used only as an indicative of mitochondrial function and only help in determination of toxicity mechanisms. But, other confirmation tests including intrinsic and extrinsic pathways are required.
